# The Multispecies Probiotic Effectively Reduces Homocysteine Concentration in Obese Women: A Randomized Double-Blind Placebo-Controlled Study

**DOI:** 10.3390/jcm9040998

**Published:** 2020-04-02

**Authors:** Karolina Majewska, Matylda Kręgielska-Narożna, Hieronim Jakubowski, Monika Szulińska, Paweł Bogdański

**Affiliations:** 1 Department of Treatment of Obesity, Metabolic Disorders and Clinical Dietetics. University of Medical Sciences in Poznań, Szamarzewskiego Str. 84, 60-569 Poznan, Poland; k.andrzejewska84@wp.pl (K.M.); mszulinska1@wp.pl (M.S.); pawelbogdanski73@gmail.com (P.B.); 2 Department of Microbiology, Biochemistry and Molecular Genetics, Rutgers University, New Jersey Medical School, International Center for Public Health, 225 Warren Street, Newark, NJ 07103-3535, USA; jakubows@njms.rutgers.edu; 3 Department of Biochemistry and Biotechnology, University of Life Sciences, Dojazd Str. 11, 60-632 Poznan, Poland

**Keywords:** homocysteine, probiotic, cardiovascular diseases, obesity

## Abstract

Dysregulated metabolism of homocysteine (Hcy) is associated with obesity. Supplementation with probiotics can potentially be a natural therapeutic method for metabolic disorders. The precise mechanism in which microbiota affect Hcy metabolism in obese individuals is still unknown. The aim of this study was to evaluate the effects of a 12-week supplementation with a multispecies probiotic on Hcy levels, oxidative stress, inflammation and lipid profile in obese patients. This randomized double-blind placebo-controlled trial was performed on 50 obese women (aged 45–70 years). Subjects were randomly assigned to take either a multispecies probiotic supplement (*n* = 25) or placebo (*n* = 25) for 12 weeks. The probiotic contained nine bacterial strains containing 2.5 × 10^9^ CFU/g. Biochemical and anthropometric measurements were carried out at baseline and after 12 weeks of intervention. At the end of the study, a significant decrease in Hcy, tumor necrosis factor α (TNF-α), total cholesterol (TC), low-density lipoprotein cholesterol (LDL) and triglyceride (TG) levels were observed in the probiotic group. The amelioration of total antioxidant status (TAS) was also observed. The 12-week supplementation of the multispecies probiotic (Ecologic^®^ BARIER) effectively reduced Hcy concentration, oxidative stress and inflammation, and improved the lipid profile. These multidirectional effects can potentially reduce cardiometabolic risks.

## 1. Introduction

Obesity is a common condition that creates a heavy social burden worldwide. The World Health Organization (WHO) reports that 39% of adults aged 18 and over were overweight in 2016 and 13% were obese [1]. Excessive body fat is linked to cardiovascular diseases [2]. The challenge for modern medicine is to develop new methods for the prevention and treatment of obesity and its complications. Healthy diet and lifestyle are important in reducing cardiovascular risk. Limiting products containing B vitamins, consuming excessive amounts of methionine-rich food, and the use of stimulants such as nicotine and caffeine can increase levels of homocysteine (Hcy) [3,4], an independent risk factor for coronary heart disease, venous thrombosis and stroke [5]. Elevated Hcy reduces the synthesis and bioavailability of nitric oxide, increases the proliferation of myocytes, adversely affects the tension of blood vessel walls and contributes to their remodeling and stiffening. These mechanisms contribute to the destruction of vascular endothelial cells and cardiovascular disease (CVD) [3,5,6,7]. New methods to lower serum Hcy levels and reduce the inflammation of endothelial cells in obese individuals are being sought. A promising solution is supplementation with probiotic bacteria. Changes in the composition of the digestive tract flora contribute to many diseases [8]. Disorders in the physiological composition of microflora impair the intestinal wall and lead to the so-called leaky gut syndrome, in which bacterial antigens penetrate the intestinal wall and cause subclinical inflammation [9]. High-fat diets consumed by obese people result in an increased number of Gram-negative bacteria in the gastrointestinal tract. The cell membranes of these bacteria contain a lipopolysaccharide (LPS) that activates Toll-like receptor 4 (TLR4), inducing endotoxemia. Chronic inflammation contributes to the development of insulin resistance, oxidative stress, dyslipidemia, increased glucose concentration and production of pro-inflammatory cytokines. This leads to damage and deterioration of the vascular endothelial function and an increased risk of CVD [10,11,12]. Normalization of the intestinal flora composition with probiotics improves the tightness of the intestinal barrier, reduces the migration of pathogenic flora to the intestinal lumen and reduces toxemia and associated inflammation. Probiotics also have antioxidant properties, improve insulin sensitivity, and have a positive effect on glucose and lipid metabolism [13,14,15,16,17]. Although multiple effects of probiotics have been studied, how probiotic supplement treatment affects Hcy levels, which may be responsible for pathological complications in obese postmenopausal women, is not known. Accordingly, the purpose of the present study was to evaluate the effect of a 12-week supplementation of the multispecies probiotic Ecologic^®^ BARIER on Hcy concentration (primary endpoint) and typical obesity complications such as oxidative stress, lipid disorders and chronic inflammation assessed by biochemical parameters (secondary endpoints).

## 2. Materials and Methods

### 2.1. Study Population

This randomized double-blind study was approved by The Bioethics Committee at Poznan University of Medical Science (No. 659/16). The study was performed in accordance with the Good Clinical Practice standards. Participants were included once they signed the informed consent. The inclusion criteria were: a signed informed consent, age between 45–70 years old, female gender, BMI 30–45 kg/m², waist circumference (WC) > 80 cm and stable body weight (±1 kg) in the month prior to the trial. Exclusion criteria were: secondary form of obesity, gastrointestinal diseases, diabetes, chronic kidney disease (GFR < 60 mL/min/1.73 m²), clinically significant impaired liver function, dyslipidemia and arterial hypertension that required pharmacological treatment in the last 3 months before the trial, clinically significant acute chronic inflammatory process, nicotine or alcohol abuse, significant changes of physical activity during the study, the use of antibiotics in the month prior to the trial, vegetarian dietary habits, the use of probiotics or prebiotics and products with a high content of dietary fiber (>400 g/daily) or a significant amount of fermented food, the use of dietary supplements in the month prior to the trial (including coffee, tea, vitamin B preparations affecting the homocysteine concentration), hormone replacement therapy or any other conditions that could be in the opinion of the investigator unsafe for participants or that could influence the effectiveness of the trial.

### 2.2. Study Design

A total of 123 obese female patients were recruited; 54 participants met the inclusion criteria and were invited to participate in the study. The sample size calculation was done based on primary outcome (homocysteine) considering 80% power at α = 0.05 [18,19]. Ultimately, 50 women participated in the study. They were allocated to either a probiotic treatment group (*n* = 25) or a placebo group (*n* = 25). All participants completed the 12-week study. After providing written, informed consent, participants underwent a physical examination and medical history was taken. Anthropometric measurements such as body mass index (BMI), waist circumference (WC), body weight and height were recorded. Resting seated blood pressure was measured three times and the average value was calculated. Participants were instructed to take the treatment (probiotic preparation or placebo) regularly and to maintain their normal diet and physical activity during study. They were asked not to consume any other probiotic-containing products, dietary supplements and antibiotics. Patients were asked to return every two weeks to monitor their compliance. At the beginning and at the end of intervention biochemical parameters were evaluated.

### 2.3. Probiotic and Placebo

Probiotic Ecologic^®^ BARRIER and placebo were prepared by Winclove Probiotics, Amsterdam, The Netherlands. Probiotic bacteria, in an amount of 2.5 × 10^9^ CFU/g, were suspended in a carrier material of corn starch and maltodextrin. The probiotic preparation contained the following bacterial strains: *Bifidobacterium bifidum* W23, *Bifidobacterium lactis* W51, *Bifidobacterium lactis* W52, *Lactobacillus acidophilus* W37, *Lactobacillus brevis* W63, *Lactobacillus casei* W56, *Lactobacillus salivarius* W24, *Lactococcus lactis* W19 and *Lactococcus lactis* W58. The placebo was not different from the probiotics in color, taste or smell but it did not contain probiotic bacteria. Participants consumed two sachets (4 g) per day for 12 weeks. The powder was dissolved in water at room temperature, left to stand for one minute and mixed before consumption. The preparation was taken on an empty stomach, either in the morning before eating and in the evening at least two hours after a meal.

### 2.4. Biochemical Analyses

Blood samples were drawn in the morning, after overnight fast, at least 12 h after the last meal, at baseline and after 12 weeks of intervention. Plasma Hcy (primary endpoint) was measured using an enzyme-linked immunosorbent assay (ELISA) with reagents from Axis-Shield Diagnostics Ltd., UK. The concentration of tumor necrosis factor α (TNF-α) was measured using ELISA with reagents from DRG Instruments GmbH, Marburg, Germany and total antioxidant status (TAS) was measured with reagents from Randox Laboratories, Ltd., Crumlin, UK by a calorimetric method. Plasma total cholesterol (TC), low-density lipoprotein (LDL) cholesterol, high-density lipoprotein (HDL) cholesterol and triglycerides (TG) were measured by a biochemical system Dimension ^®^ (Siemens, Newark, NJ, USA).

### 2.5. Statistical Analysis

Data are shown as mean ± SD. Changes in the values of the parameters examined due to probiotic supplementation and a placebo are presented in the form of delta (Δ) values. The Shapiro–Wilk test was used to assess normality of distribution. The comparative analysis of the parameters between the probiotic group and the placebo group, which showed compliance with the normal distribution, was performed with the paired, two-tailed Student’s *t*-test. The non-parametric Mann–Whitney U test was used for unrelated variables that did not show compliance with normal distribution. The results before supplementation were compared with the results obtained after the end of the 12-week intervention in both groups using the Wilcoxon signed-rank test, when the parameters were not compatible with the normal distribution, and using the paired, two-tailed Students’ *t*-test when such compliance was demonstrated. The correlation of the parameters (Δ) in the probiotic group was determined using the Spearman rank correlation test. A multiple linear regression analysis of Hcy concentration dependence on TC, HDL, LDL-cholesterol, TG, TNF-α and TAS level before and after probiotic supplementation was done. Statistical calculations were made using the Statistica 13.1 software (StatSoft^®^ Cracow, Poland). The results were considered significant at a value of *p* < 0.05.

## 3. Results

**Figure 1:**
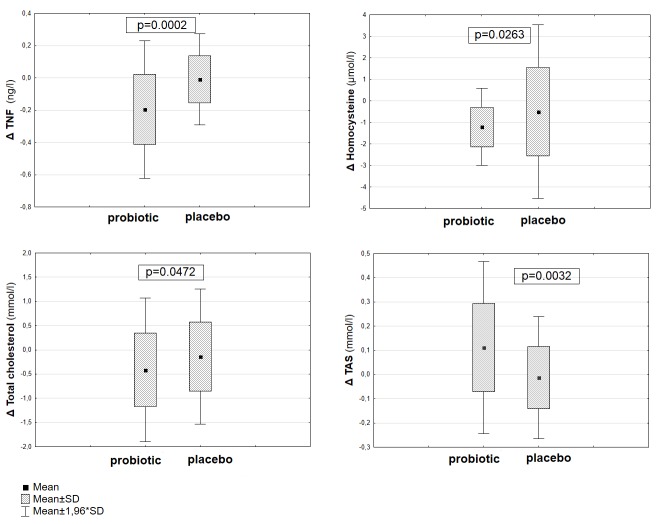
Comparative analysis of the change (Δ) in Hcy, tumor necrosis factor α (TNF-α), total cholesterol (TC) and total antioxidant status (TAS) concentration between groups.

The anthropomorphic characteristics of the probiotic and placebo groups included in the study are shown in Table 1. At baseline, the two groups did not show any statistically significant differences in the values of the examined parameters; moreover, there were no significant changes in anthropometric parameters and blood pressure values between and within groups observed at the end of the study. The 12-week probiotic supplementation resulted in a significant decrease of Hcy (*p* < 0.0001), TNF-α (*p* = 0.0001), TC (*p* = 0,0020), LDL (*p* = 0.0149), TG (*p* = 0.0140) and TAS (*p* = 0.0076) levels compared to the baseline values (Table 2). In contrast, no significant differences in Hcy, TNF-α, TAS and lipid profile were found after the trial in the placebo group (Table 2). Comparative analysis showed that the changes (Δ) of Hcy (*p* = 0.026), TNF-α (*p* = 0.0002), TC (*p* = 0.047) and TAS (*p* = 0.003) concentration after 12 weeks were statistically significant in the probiotic group compared to the placebo group. Comparative analysis of TNF-α (*p* = 0.0070) concentration and TAS level (*p* = 0.0017) at the end of the intervention period demonstrated a statistically significant difference between groups (Figure 1, Table 3).

Correlation analysis was performed for the group supplementing with the probiotic preparation to assess the relationship between the change (Δ) in Hcy concentration and the change (Δ) in TC, LDL, HDL, TG, TNF-α and TAS levels. The analysis did not show any statistically significant correlations (Table 4). The multiple linear regression analysis showed that the change in Hcy was not associated with the changes in lipid profile, TNF-α and TAS levels in the probiotic group (Table 5).

## 4. Discussion

Much emphasis has recently been placed on natural means of preventing and treating disease. Patients seek functional foods and supplements that could be useful in treating obesity and its comorbidities. In recent years there has been a very large increase in the consumption of dietary supplements in Poland [20]. Probiotic supplements are receiving more and more attention due to their potential cardioprotective effects [21,22]. In this study, we have shown that 12-week probiotic supplementation in obese women resulted in a significant reduction in Hcy concentration. The reduction in Hcy concentration may result from the synthesis of folates, vitamin B6 and vitamin B12, cofactors of Hcy-metabolizing enzymes, by the bacterial flora of the gastrointestinal tract. The ability of bacteria to produce folic acid is strain-dependent. This potential is demonstrated by lactic acid bacteria, such as *Lactococcus lactis*, *Streptococcus thermophilus* and *Lactobacillus plantarum*, as well as bacteria belonging to the *Bifidobacterium* species [23,24]. Hyperhomocysteinemia often co-occurs with disturbances in lipid metabolism. Probiotic bacteria can effectively reduce cardiovascular risks by lowering Hcy levels and improving lipid profile parameters. There are several mechanisms postulated by means of which the microflora influences the lipid profile. These include participation of bacteria in the synthesis and transformation of bile acids into secondary bile acids and a reduction of cholesterol to coprosterol in the gastrointestinal tract. Bacteria use cholesterol and bile acids to build their own cell membranes [25]. Based on the analysis of available studies, Ivey et al. concluded that the hypocholesterolemic effect of probiotic supplementation depends on the initial TC concentration [25].

In the present study, we have shown that 12-week probiotic supplementation in obese women led to improvement of the lipid profile. Numerous studies show that there is a relationship between Hcy concentration and lipid profile disorders [26,27]. Hcy initiates the formation of oxidized low-density lipoproteins (oxLDL), which strongly damage vascular endothelial cells. Moreover, it reduces the formation of HDL cholesterol by inhibiting the synthesis of ApoA-I in the liver [28,29,30]. Qujeq D. et al. showed that patients with myocardial infarction had higher serum Hcy levels than patients in the control group (*p* < 0.05) and that there was a significantly negative correlation between Hcy concentration and HDL cholesterol concentration (*p* < 0.05, r = −0.93) and a positive correlation between Hcy concentration and LDL cholesterol (*p* < 0.05, r = 0.98) [26]. However, in the present study we have not found any significant relationship between the change (Δ) in Hcy and the lipid parameters in the group receiving probiotic supplementation. The beneficial effects of probiotic bacteria are related to the type and amount of bacteria included in the supplemented probiotic preparation. It is possible that if the supplementation of probiotic bacteria in the present study was conducted for a longer time period, statistically significant correlations would be obtained. Obesity and hyperhomocysteinemia induce chronic inflammation and oxidative stress [5,31] and there is a significant positive correlation between Hcy and TNF-α concentration in groups of hypertensive patients (r = 0.48; *p* < 0.0001) [32]. Due to their immunomodulatory properties, probiotic bacteria are involved in the immune response of the host and they reduce inflammation, the expression of pro-inflammatory cytokines and oxidative stress [14]. The 12-week probiotic supplementation significantly decreased TNF-α concentration and increased TAS level. However, the correlation analysis performed in the group using probiotic supplementation between the change (Δ) in Hcy concentration and the change (Δ) in TNF-α and TAS concentration did not reach statistical significance. The effect of probiotic bacteria on inflammation parameters and oxidative stress is related to the type and amount of bacteria included in the supplemented probiotic preparation. Further research needs to be conducted to identify bacterial strains that are characterized by the highest antioxidant and immunomodulatory potential and to determine the minimum time of supplementation necessary to obtain optimal therapeutic effects.

To determine whether the changes in Hcy concentration caused by probiotic treatment are responsible for changes in other variables, we performed a multiple linear regression analysis. However, we found that changes in Hcy were independent of the other variables. This suggests that the probiotics simultaneously influenced Hcy concentration and other tested parameters. The novelty of this work was based on the studied population and the specific, complex composition of the probiotic used in this trial. The beneficial effects of supplementation with probiotic bacteria on the Hcy levels, lipid profile, oxidative stress and inflammation demonstrated in the present study, along with results obtained by other investigators, are summarized in Table 6 [33,34,35,36]. Taken together, these findings suggest that probiotics can serve as a natural therapeutic method for reducing cardiovascular risk.

## 5. Conclusions

Probiotics have been widely known and used for many years. Evidence from published studies indicates their importance in the treatment and prophylaxis of metabolic disorders, obesity and CVD. The results of this study indicate that a 12-week supplementation of the multispecies probiotic Ecologic^®^ BARRIER effectively reduced Hcy concentration, oxidative stress and inflammation, and improved lipid profile. By influencing these risk factors, probiotic bacteria show multidirectional effects and reduce cardiometabolic risks. Thus, a multispecies probiotic can be considered as an additional therapy in obesity to reduce cardiovascular risk factors.

## Figures and Tables

**Table 1:** Characteristics of the probiotic group and the placebo group at the beginning of the study.

Characteristics	Probiotic(*n* = 25)	Placebo (*n* = 25)	*p*-Value
Age (years)	55.2 ± 6.9	58.7 ± 7.3	NS
Weight (kg)	94.5 ± 16.6	92.8 ± 11.9	NS
Height (cm)	160.8 ± 6.2	160.4 ± 6.4	NS *
BMI (kg/m^2^)	36.6 ± 6.0	36.1 ± 4.4	NS
Waist circumference (cm)	109.8 ± 11.7	109.9 ± 8.3	NS *
SBP (mmHg)	134.8 ± 10.1	133.6 ± 12.2	NS
DBP (mmHg)	79.9 ± 8.1	83.8 ± 7.3	NS *

BMI, body mass index; SBP, systolic blood pressure; DBP, diastolic blood pressure; NS, not significant; * Student’s *t*-test.

**Table 2:** Comparative analysis of the Hcy, TNF-α, TAS level and the lipid profile parameters in the probiotic and placebo group before and after intervention.

Variables	Probiotic (*n* = 25)			Placebo (*n* = 25)		
	Before	After	*p*-Value	Before	After	*p*-Value
Hcy (µmol/L)	11.32 ± 2.23	10.11 ±1.57	<0.0001	11.63 ± 2.08	11.13 ± 2.13	NS *
TNF-α (ng/L)	1.04 ± 0.34	0.85 ± 0.23	0.0001	1.05 ± 0.34	1.04 ± 0.31	NS
TAS (mmol/L)	1.65 ± 0.20	1.76 ± 0.14	0.0076	1.65 ± 0.20	1.64 ± 0.12	NS
TC (mmol/L)	5.65 ± 0.85	5.24 ± 0.80	0.0020	5.27 ± 0.91	5.12 ± 0.98	NS *
LDL (mmol/L)	3.09 ± 0.82	2.97 ± 0.96	0.0149	3.00 ± 0.87	2.93 ± 0.91	NS *
HDL (mmol/L)	1.36 ± 0.28	1.41 ± 0.22	NS *	1.35 ± 0.26	1.44 ± 0.28	NS *
TG (mmol/L)	1.86 ± 0.88	1.73 ± 0.63	0.0140	1.60 ± 0.71	1.53 ± 0.78	NS

TNF-α-tumor necrosis factor α; TAS-total antioxidant status; TC-total cholesterol; LDL-low density lipoprotein; HDL-high density lipoprotein; TG-triglycerides; Hcy-homocysteine; * Students’ *t*-test.

**Table 3:** Comparative analysis of Hcy, TNF-α, TAS level and the lipid profile parameters between the probiotic group and the placebo group.

Probiotic vs. Placebo			
Variables	*p*-Value		
	Before	After	Δ
Hcy(µmol/L)	NS	NS *	0.0263
TNF-α(ng/L)	NS	0.0070	0.0002
TAS (mmol/L)	NS	0.0017 *	0.0032
TC (mmol/L)	NS	NS	0.0472
LDL (mmol/L)	NS *	NS	NS
HDL (mmol/L)	NS *	NS *	NS *
TG (mmol/L)	NS	NS	NS

Δ, delta; TNF-α, tumor necrosis factor α; TAS, total antioxidant status; TC, total cholesterol; LDL, low density lipoprotein; HDL, high density lipoprotein; TG, triglycerides; Hcy, homocysteine; * Student’s *t*-test.

**Table 4:** Correlation analysis in the probiotic group between the change (Δ) in Hcy concentration and the change (Δ) in TC, LDL, HDL-cholesterol, TG, TNF-α and TAS level.

	Δ Hcy (µmol/L)	RP-Value
Δ TNF-α (ng/L)	0.36	NS
Δ TAS (mmol/L)	0.12	NS
Δ TC (mmol/L)	0.15	NS
Δ LDL (mmol/L)	0.29	NS
Δ HDL (mmol/L)	0.02	NS
Δ TG (mmol/L)	−0.17	NS

TNF-α, tumor necrosis factor α; TAS, total antioxidant status; TC, total cholesterol; LDL, low density lipoprotein; HDL, high density lipoprotein; TG, triglycerides; Hcy, homocysteine.

**Table 5:** Multiple linear regression analysis of Hcy concentration dependence on TC, HDL, LDL-cholesterol, TG, TNF-α and TAS level before and after probiotic supplementation.

Multiple Linear Regression, Dependent Variable Hcy (µmol/L)						
	Before Probiotic Suplementation			After Probiotic Supplementation		
	β	Standard Error of β	*p*-Value	β	Standard Error of β	*p*-Value
Intercept	12.20	7.60	NS	13.70	6.76	NS
TNF-α (ng/L)	0.44	1.68	NS	1.09	1.87	NS
TAS (mmol/L)	1.45	3.12	NS	−1.82	2.78	NS
TC (mmol/L)	−0.01	0.02	NS	0.00	0.02	NS
LDL (mmol/L)	0.00	0.02	NS	0.00	0.01	NS
HDL (mmol/L)	−0.02	0.07	NS	−0.02	0.07	NS
TG (mmol/L)	0.00	0.01	NS	0.00	0.01	NS
R	0.23			0.27		
R^2^	0.06			0.07		
F(6,18)	0.18			0.24		

TNF-α, tumor necrosis factor α; TAS, total antioxidant status; TC, total cholesterol; LDL, low density lipoprotein; HDL, high density lipoprotein; TG, triglycerides; Hcy, homocysteine.

**Table 6:** Summary of studies analyzing the effect of probiotic supplementation on Hcy levels, lipid profile, oxidative stress and inflammation [31,32,33,34,35].

Author	Study Group	Probiotics	Study Duration	Dosage	Results
Valentini et al. [33]	Healthy adults (65–85 years)	*B. infantis* DSM24737, *B. longum* DSM24736, *B. breve* DSM24732, *L. acidophilus* DSM24735, *L. delbrueckii ssp*. *bulgaricus* DSM24734, *L. paracasei* DSM24733, *L. plantarum* DSM24730, *S. thermophilus* DSM24731	56 days	112 × 10^9^ CFU/caps 2 caps/daily	Hcy↓ vitamin B12, folic acid↑ No significant effect on inflammatory biomarkers, lipid profile
Rajkumar et al. [34]	Overweight, healthy adults (40–60 years)	*B. longum*, *B. infantis*, *B. breve*, *L. acidophilus*, *L. paracasei*, *L. delbrueckii spp. bulgaricus*, *L. plantarum*, *S. salivarius spp. Thermophilus*	6 weeks	112.5 × 10^9^ CFU/caps	HDL ↑ TC, TG, LDL, VLDL↓ hs-CRP↓
Vagher-Mehrabany et al. [35]	Women with rheumatoid arthritis (20–80 years)	*Lactobacillus casei* 01	8 weeks	10^8^ CFU/caps	Il-6, Il-12, TNF-α,↓ Il-10 ↑ No significant effect on Il-1β
Asemi et al. [36]	Diabetic patients (35–70 years)	*L. acidophilus*, *L.casei*, *L. rhamnosus,* *L. bulgaricus,* *B. breve,* *B. longum,* *S. thermophilus*, Fructo-oligosaccharide.	8 weeks	2 × 10^9^ CFU 7 × 10^9^ CFU 1.5 × 10^9^ CFU 2 × 10^8^ CFU 2 × 10^10^ CFU 7 × 10^9^ CFU 1.5 × 10^9^ CFU 100 mg/caps	GSH, TAC, LDL↑ hs-CRP, HDL ↓ No significant effect on TC, TG,
Barreto et al. [18]	Postmenopau-sal women with metabolic syndrome	*L. plantarum*	90 days	1.25 × 10^7^ CFU/g (fermented milk–80 mL/d)	Hcy, TC, Il-6↓ No significant effect on HDL, LDL, TG, CRP, TNF-α
Present study	Obese women (45–65 years)	Probiotic Ecologic^®^ BARRIER	12 weeks	2.5 × 10^9^ CFU/g	Hcy, TC, TNF-α↓ TAS↑

TC, total cholesterol; LD, low density lipoprotein; HDL, high density lipoprotein; VLDL, very low density lipoprotein; TG, triglycerides; Hcy, homocysteine, Il-1β, interleukin 1β; Il-6, interleukin 6; Il-12, interleukin 12; Il-10, interleukin 10; TNF-α, tumor necrosis factor α; hs-CRP, high-sensitivity C-reactive protein; GSH, reduced glutathione; TAC, total antioxidant capacity.
